# Performance and Limitations of Nickel‐Doped Chromite Anodes in Electrolyte‐Supported Solid Oxide Fuel Cells

**DOI:** 10.1002/cssc.202100330

**Published:** 2021-05-07

**Authors:** Matthias Riegraf, Diana M. Amaya‐Dueñas, Noriko Sata, K. Andreas Friedrich, Rémi Costa

**Affiliations:** ^1^ Institute of Engineering Thermodynamics German Aerospace Center (DLR) Pfaffenwaldring 38–40 70569 Stuttgart Germany; ^2^ Institute for Building Energetics Thermotechnology and Energy Storage University of Stuttgart Pfaffenwaldring 31 70569 Stuttgart Germany

**Keywords:** electrocatalysis, exsolution, fuel cell, perovskite, solid oxide cells

## Abstract

Ni‐doped chromite anodes were integrated into electrolyte‐supported cells (ESC) with 5×5 cm^2^ size and investigated in fuel cell mode with H_2_/H_2_O fuel gas. Both a stoichiometric and a nominally A‐site deficient chromite anode material showed promising performance at 860 °C approaching the ones of state‐of‐the‐art Ni/Gd‐doped ceria (CGO) anodes. While the difference in polarization resistance was small, an increased ohmic resistance of the perovskite anodes was observed, which is related to their limited electronic conductivity. Increasing the chromite electrode thickness was shown to enhance performance and stability considerably. Degradation increased with current density, suggesting its dependency on the electrode potential, and could be reversed by redox cycling. Sulfur poisoning with 20 ppm hydrogen sulfide led to rapid voltage drops for the chromite anodes. It is discussed that Ni nanoparticle exsolution facilitates hydrogen dissociation to the extent that it is not rate‐limiting at the investigated temperature unless an insufficiently thick electrode thickness is employed or sulfur impurities are present in the feed gas.

## Graphical abstract

**Appearing from nowhere**: Alternative perovskite anodes for solid oxide fuel cells show the exsolution of anchored Ni nanoparticles under reducing conditions. In the present work, their performance and durability is compared to commercial electrodes.

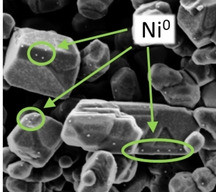

## Introduction

Although their lifetime and performance are continuously improved, solid oxide fuel cells (SOFC) keep on struggling with commercialization. A key factor determining the economic viability of solid oxide fuel cells is their durability, which, despite continuous lifetime and performance increase over the last decades, is currently restricted by degradation of the different cell components, especially the electrode materials.

An ideal SOFC fuel electrode (anode) shows high electronic and ionic conductivity, good thermal and chemical stability with the adjacent layers under reducing conditions and excellent (electro‐)catalytic activity towards hydrogen and hydrocarbon oxidation. In addition, high resistivity towards redox cycling, carbon deposition and sulfur poisoning during operation with hydrocarbons or reformates is desirable.

Ni‐based cermet anodes such as Ni/Yttria‐stabilized zirconia (YSZ) have been developed where the metallic Ni provides the electronic conductivity and the YSZ phase, which is also the electrolyte material, provides the ionic conductivity. Ni/YSZ anodes show excellent performance during hydrogen oxidation, water gas shift reaction and reforming of various fuels such as methane due to the high catalytic activity of the Ni phase. These advantages and the outstanding mechanical stability of YSZ have made Ni/YSZ the historical electrode of choice in commercial applications of the anode‐supported cell (ASC) architecture. However, despite its advantages, the electrode also faces degradation problems such as low tolerance towards sulfur impurities in the feed gas, susceptibility towards carbon formation and poor redox stability. Ni/Gadolinium‐doped ceria (CGO) electrodes have shown lower short‐term performance drops and increased long‐term stability upon sulfur exposure, also in reformate fuels.^[1]^ Furthermore, there are reports indicating lower overpotentials and increased tolerance towards carbon formation and increased redox stability.^[2]^ These favorable characteristics are related to the mixed ionic and electronic conductivity (MIEC) properties of CGO at high temperatures and in reducing atmospheres,^[3]^ which extends the active reaction zone from the triple‐phase boundary (TPB) to the double‐phase boundary between CGO and gas phase.^[4]^ Despite the good inherent (electro‐) catalytic activity of CGO and its decent electronic conductivity, both properties are still significantly enhanced by adding a metallic Ni phase to the electrodes. Therefore, redox cycling of conventional sintered Ni/CGO electrodes is still an issue due to the dimensional expansion of Ni upon oxidation, which can lead to Ni grain coarsening and even electrode delamination.^[2c]^ In an attempt to increase dimensional electrode stability, infiltration of Ni nanoparticles into a ceramic backbone has shown to lead to outstanding electrode performance, but rapid grain coarsening at higher temperatures entails high degradation rates.^[5]^ Furthermore, mechanical stability of Ni/CGO is rather poor, which currently restricts their use to electrolyte‐supported cell (ESC) and metal‐supported cell architectures.^[6]^

As alternative fuel electrode materials, a variety of single‐phase perovskite materials (ABO_3_) that display desirable MIEC properties have been investigated over the last decades. To ensure high phase stability the host B‐site cation should remain in an oxidized state at low *p*O_2_ to withstand reduction to its metallic form and the associated decomposition of the perovskite structure when exposed to hydrogen. Most frequently, Cr, Mn, and Ti cations are employed as host B‐site cations.^[7]^ Electrical conductivity and electrocatalytic activity can be tuned by partial substitution with a wide variety of multivalent dopants of the A and B cations. For example, (electro‐)catalytic activity can be significantly improved by introduction of transition metals such as Ni,^[8]^ Ru,[^8a^, ^9^] Pd^[10]^ on the B site, which are released (exsolved) on the surface as metal nanoparticles upon reduction. This process occurs since the lattice loses oxygen and gains electrons upon reduction until metal nucleation becomes favorable. The nanoparticles show high stability due to their strong anchorage in the perovskite matrix, and thus, higher resistance against particle agglomeration than infiltrated nanoparticles.^[8c]^ Moreover, such electrodes have shown excellent redox stability with regeneration of nanoparticles through redox cycling.[^8a^, ^8e^, ^10a^]

LaCrO_3_‐based oxides have been investigated as interconnect materials for SOFCs and are considered as potential anode materials due to their high electronic conductivity and stability in both reducing and oxidizing conditions at high temperatures and also due to their low activity toward carbon deposition.^[11]^

For stoichiometric perovskites, Sr was found to be a particularly good choice as A‐site substituent leading to high stability of the perovskite structure and high electrical conductivity.[^11b^, ^12^] Optimum electrical conductivity can be achieved for 30–40 % Sr on the A‐site.^[13]^ Furthermore, Sr substitution on the A‐site leads to a better match of the coefficient of thermal expansion (CTE) with CGO.^[11a]^ Among a multitude of different B‐site substituents, Ni was identified as very promising leading to excellent (electro)catalytic activity.[^11b^, ^12a^] Additionally, Ni‐doped LaCrO_3_‐based oxides also showed lower activity towards carbon formation than Ni/YSZ cermet anodes.^[14]^ Based on such considerations, Kobsiriphat et al. investigated La_0.8_Sr_0.2_Cr_1‐*y*_X_*y*_O_3‐*δ*_ (X=Ni, Ru) electrodes and observed Ni nanocluster formation of approximately 10 nm in diameter upon reduction but also considerable coarsening to 50 nm after 300 h at 800 °C.^[8a]^

Recent studies on titanates have revealed that the exsolution process is favored in A‐site deficient perovskites, which can lead to a higher concentration of Ni nanoparticles on the perovskite surface since exsolution acts to locally revert the perovskite towards a “defect‐free” ABO_3_ stoichiometry.[^8b^, ^8c^, ^15^] However, such titanate electrodes with Ni exsolution have mainly been investigated on button cell level (≈1 cm^2^ active area) and have not been directly compared to state‐of‐the‐art Ni/cermet electrodes in operation with equivalent boundary conditions.

Furthermore, only one pioneering study in literature has investigated A‐site deficient lanthanum chromites and their application in SOFC: Sun et al. observed increased performance of an A‐site deficient La_0.6_Sr_0.3_Cr_0.85_Ni_0.15_O_3‐*δ*_ anode in comparison to the stoichiometric perovskite electrode (La_0.7_Sr_0.3_Cr_0.85_Ni_0.15_O_3‐*δ*_) in small button cells at 800 °C.^[8d]^ Significant performance degradation was observed within 24 h of operation related to Ni particle coarsening, but redox cycling was shown to lead to full recovery. Interestingly, a promoting effect of 5000 ppm H_2_S on hydrogen oxidation was reported.

Recently, we have introduced the chromite formulation La_0.65_Sr_0.3_Cr_0.85_Ni_0.15_O_3‐*δ*_ (L65SCrN) and demonstrated the successful up‐scaling of exsolution‐based perovskite electrodes from the commonly used button cell level to 5×5 cm^2^ large ESC.^[16]^ The performance of the cell in (co‐)electrolysis mode was promising. In the present study, cells with this perovskite electrode are investigated in fuel cell operation. The aim of the study is to assess electrode performance and durability of nominally A‐site deficient L65SCrN and stoichiometric L70SCrN electrodes and their comparison with state‐of‐the‐art Ni/CGO electrodes.

## Results and Discussion

In the following subsections, an analysis of the crystallographic structure of L65SCrN and L70SCrN is shown. Then, the investigation of ESC with LSCrN electrodes with regards to performance, durability, redox stability, and finally towards their sulfur tolerance will be presented.

### Crystallographic structure analysis

**Figure 1:**
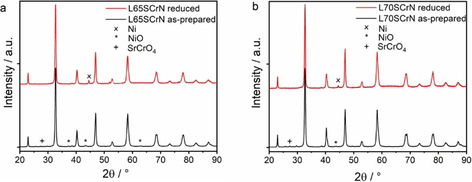
XRD patterns of (a) L65SCrN and (b) L70SCrN powder. Both (a) and (b) show as‐prepared and reduced (5 % H_2_/95 % N_2_ at 1000 °C for 1 h) samples.

The crystalline structures of both commercial L70SCrN and L65SCrN ceramic powders were analyzed by X‐ray diffraction (XRD). The as‐prepared and the reduced powders were analyzed in order to check the crystalline phases. All X‐ray diffractograms are shown in Figure 1. The main phase is identified as an LSCrN perovskite structure, consistent with the ones observed in our previous study (PDF# 01‐076‐7024).^[16]^

Furthermore, both powders show a secondary SrCrO_4_ phase that cannot be detected anymore after reduction. This is consistent with literature studies that reported the formation of SrCrO_4_ after the annealing of chromites at high temperature in air.^[17]^ Since no SrCrO_3_ signal was observed after reduction, it is likely that SrCrO_4_ formation was reversed under reducing conditions and the cations were integrated into the perovskite structure. This can be explained by the partial oxidation of Cr^3+^ species in the perovskite lattice to Cr^6+^ and the segregation of SrCrO_4_ at the surface. In literature reports, Cr^6+^ disappeared gradually during reduction in H_2_/H_2_O at 700 °C, and after reduction the surface was dominated by Sr^2+^ and Cr^3+^ cations.^[17b]^ Thus, it is likely that the observed SrCrO_4_ on the two powders is due to the surface chemistry of the perovskite grains that differs from the bulk in oxidizing conditions.

In both oxidized powders, a NiO secondary phase could be detected. A magnification of the XRD patterns of the relevant angles is depicted in Figure S2 and shows that the NiO signal at the characteristic diffraction angles of approximately 37 and 42° is significantly stronger in the L65SCrN than in the L70SCrN sample. The amount of NiO in the L7SCrN perovskite was determined to be only 0.75 % by Rietveld refinement (Supporting Information) and thus, it is assumed that the crystal structure is indeed stoichiometric (or close to). In the L65SCrN powder, the NiO concentration was 4.55 %. Based on its nominal perovskite composition and the amount of NiO, the real cationic between A‐ and B‐site of L65SCrN is 0.995. Thus, the composition of the perovskite phase was likely close to a stoichiometric one with an estimated A‐site deficiency of <1 %. The difference in La content in the supplied powders likely leads to a different La/Sr ratio that influences the electrode properties. In fact, the small shift of the perovskite peak at approximately 40° to a lower diffraction angle for the L65SCrN demonstrates that the chemical compositions of two perovskites are different. In the following, L65SCrN denotes a composite of a perovskite with A‐site deficiency and reduced NiO.

After reduction, only metallic nickel could be identified as secondary phase. It is reasonable to consider that the metallic Ni phase in the reduced samples stems from both the initial NiO secondary phase and the exsolved Ni from the perovskite lattice. The observed Ni diffraction peak is rather sharp suggesting relatively large particles sizes over 100 nm. Diffraction peaks of Ni nanoparticles could be hidden in the background in this case when the amount of nanoparticles is not very high. Based on the quantified amount of NiO from the Rietveld refinement, an estimate of the TPB lengths between reduced Ni, perovskite and gas phase for both the reduced NiO and exsolved Ni was carried out and showed that the TPB length can be expected to be about one order of magnitude higher for the exsolved Ni particles due to their small particles size and, thus, dominate the electro‐catalytic activity (see the Supporting Information).

### Electrochemical performance

**Figure 2:**
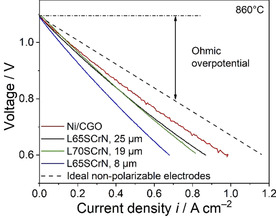
*i*–*V* curves of the tested cells at 860 °C and in 97 % H_2_, 3 % H_2_O fuel gas. The dashed curve denotes a cell with ideal non‐polarizable electrodes, that is, without polarization resistance.

Cell performance was assessed by means of current‐voltage characteristics (*i*‐*V* curves). An initial *i*‐*V* curve of all the cells tested in the present study is shown in Figure 2. The Ni/CGO‐based cell displayed the highest performance with 0.98 A cm^−2^ at 0.6 V, which is consistent with the recently observed performance of a similar cell.^[18]^ The cell with 25 μm thick L65SCrN electrode demonstrated a promising performance of 0.87 A cm^−2^ at 0.6 V approaching the one of the state‐of‐the‐art Ni/CGO‐based ESC. The performance of the cell with 19 μm thick electrode L70SCrN was slightly lower showing a current density of 0.81 A cm^−2^ at 0.6 V. The cell with 8 μm thick L65SCrN electrode displayed a significantly lower performance of 0.68 A cm^−2^ at 0.6 V.

**Figure 3:**
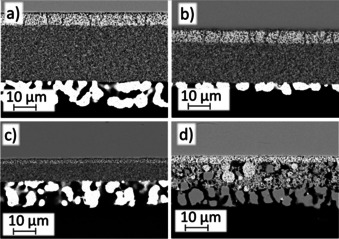
SEM cross‐section images of the anodes investigated in the present study: (a) 25 μm thick L65SCrN anode with Pt current collector layer, (b) 19 μm thick L70SCrN anode with Pt current collector layer, (c) 8 μm thick L65SCrN with Pt current collector layer, (d) Ni/CGO anode with Ni current collector layer.

Anodes of all cells are depicted in Figure 3. It can be seen that the microstructure of the different L65SCrN (Figure 3a,c) and L70SCrN (Figure 3b) anodes is similar with the same grain size of approximately 300–500 nm. Since the 25 μm thick L65SCrN and the 19 μm thick L70SCrN electrode were applied onto the substrate with the same mesh, the difference in their thickness is likely due to a slightly different viscosity of the prepared inks. The Pt particles in the current collector layers are 5–10 μm large. To investigate a possible effect of Pt on the electrochemical performance, a cell without LSCrN functional fuel electrode and only brushed Pt was investigated. The *i*‐*V* curve of the cell (Figure S1) in pure hydrogen at 860 °C shows a low maximum current density of 0.18 A cm^−2^ at 0.6 V. Thus, it can be assumed that only a small TPB length between LSCrN/Pt/gas phase with limited electro‐catalytic performance is created, and the performance differences between the various cells can be ascribed to differences in behavior of the different LSCrN electrodes. The thickness of the Ni/CGO anode including the Ni current collector is about 25 μm and thus, comparable to Figure 3b.

**Figure 4:**
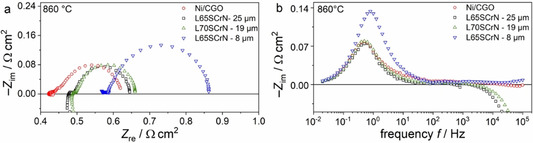
(a) Complex‐plane and (b) imaginary impedance plots of the four tested cells at 860 °C, OCV and 97 % H_2_, 3 % H_2_O.

In Figure 4a,b, initial impedance spectra of all cells are depicted at open circuit voltage (OCV). They show that the main reason for the highest performance of the Ni/CGO‐based cell is the ohmic resistance of 0.42 Ω cm^2^ which is significantly lower than for the other cells. The cells with 25 μm thick L65SCrN and 19 μm thick L70SCrN anode display similar ohmic resistance values of 0.48 and 0.49 Ω cm^2^, respectively. On the contrary, the cell with 8 μm thick L65SCrN shows a considerably higher value of 0.56 Ω cm^2^.

The ohmic resistance of SOC is usually assumed to be governed by the electrolyte due to its limited ionic conductivity. However, this cannot explain the different values in the present study because all cells employed the same substrate. Since the cathode is also the same in all cells (or very similar in case of LSCF/CGO), the different ohmic resistance values must be caused by the anodes. It was shown that the ionic resistivity of the ceramic matrix in the porous Ni cermet anode structures leads to losses in the polarization resistance since it is coupled to the charge transfer reaction and porous gas diffusion, and not increased ohmic resistance.^[19]^

Therefore, it is most likely that the increased ohmic resistance of the LSCrN electrodes in comparison to Ni/CGO is rather related to the comparatively low electronic conductivity of the perovskite. In our previous work, we observed that the p‐type electronic conductivity of LSCrN decreased with decreasing *p*O_2_, suggesting that ohmic resistance losses in the electrode are particularly high at low humidity in the fuel gas.^[16]^ In this regard, the perovskite composition can be expected to have a significant influence on the electronic conductivity.^[13]^ However, possible differences between L65SCrN and L7SCrN do not lead to significantly different ohmic resistance values.

**Figure 5:**
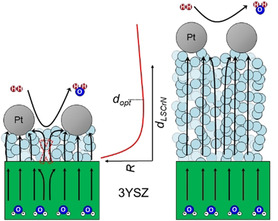
Schematic illustration of the current lines in electrolyte and LSCrN fuel electrode and the resistance *R* as function of the electrode thickness *d*_LSCrN_. *d*_opt_ denotes the optimum electrode thickness with the lowest resistance value.

Additionally, the 8 μm thick L65SCrN electrode also shows an increased polarization resistance of 0.30 Ω cm^2^ in comparison to the other cells with thicker electrodes (0.17–0.2 Ω cm^2^, see discussion below). The reason for the increased resistance values is probably the thickness of the electrode being below the optimum electrochemically active thickness *d*_opt_ as described in Ref. [20]. The electrode polarization resistance as a function of the electrode thickness is illustrated in the middle panel of Figure 5. The resistance evolution with thickness is a complex function of the geometry and material of the current collector, as well as porosity, microstructure, electronic/ionic conductivity, and electro‐catalytic activity of the electrode. In addition, the in‐plane electrode electronic conductivity and the ratio between electrode thickness and current collector spacing are low when the electrode thickness is low and this leads to the current restriction effect (left panel of Figure 5). La_1‐*x*_Sr_*x*_CrO_3_‐based electrodes have shown decent electrical conductivity under reducing conditions (0.1–10 S cm^−1^ at 800 °C).[^12b^, ^13^] Still, the electrical conductivity Ni/YSZ anodes in reducing atmosphere lies significant higher, typically above 1000 S cm^−1^.^[21]^ Thus, it is very likely that the performance of thin L65SCrN anodes are limited by the perovskite's electronic conductivity. This leads to a bending of the current lines in the electrode and heterogeneous current density distribution in the electrode as sketched in Figure 5 (left panel). In particularly pronounced cases, the current then almost exclusively flows under the current collectors. As a consequence, this can cause a fraction of the electrode and even the electrolyte to become inactive leading to a reduction in effective electrode/electrolyte area. Pouillet's law states the following [Eq. 1]:

(1) $$R=\frac{1}{\sigma }\frac{d}{A_{\mathrm{eff}}}$$

with *R* being the electrical resistance, *σ* the electrical conductivity, *d* the electrode thickness, and *A*_eff_ the effective cross‐sectional area, so the ohmic and polarization resistance increase with decreasing effective area. An electrode thickness *d*_opt_ exists, for which the ratio *d*_LSCrN_/*A*_eff_ is minimal.

A further increase of electrode thickness beyond the electrochemically active electrode thickness also leads to an additional ohmic resistance if the electronic conductivity of the electrode is low (Figure 5, right panel). Therefore, it is likely that further improvements in ohmic resistance can be achieved by optimizing electrode morphology and processing conditions, possibly reducing the cell resistance of the perovskite‐based cells closer to the ones of state‐of‐the‐art Ni/CGO cells.

Interestingly, the polarization resistance of the Ni/CGO‐based cell (0.2 Ω cm^2^) is similar or even slightly larger than the ones of the cells with 25 μm thick L65SCrN and 19 μm thick L70SCrN anodes of 0.17 Ω cm^2^, demonstrating excellent perovskite kinetics. The polarization resistance of all three cells is dominated by a semicircle with a peak frequency of 0.5 Hz. At the peak frequency and below (<1 Hz) the curves of all cells coincide, which indicates that it is probably associated with the gas conversion in agreement with what we already suggested in our recent studies.[^18^, ^22^] However, although the shape of this low frequency contribution is the same for all cells, the gas conversion resistance is generally strongly dependent on variations of the steam content at the present operating conditions (*p*H_2_O=0.03 bar), which could be caused by small deviations in sealing and/or gas flow rates. In addition, the inlet gas is humidified with a water bubbler the exact temperature of which can show small variations depending on the room temperature. Thus, minor differences between the gas conversion resistance values cannot be excluded.

Moreover, for Ni/CGO‐based cells of the same type as used in the present study, we have reported the peak frequency of the anode charge transfer process to be between 1–10 Hz.[^18^, ^23^] The impedance response at these frequencies is also increased in comparison with the cells with 25 μm thick L65SCrN and 19 μm thick L70SCrN anodes and responsible for the difference in polarization resistance between the cells. The increased polarization resistance of the cell with 8 μm thick L65SCrN electrode is mainly caused by an increased impedance response at frequencies of approximately 1 Hz. Therefore, it is concluded that the L65SCrN anode process lies at frequencies of 1–10 Hz similar to the Ni/CGO anode process. However, since anode processes and gas conversion contribution overlap in the impedance spectra, it is difficult to quantify the anode resistance.

Moreover, the cell resistance at OCV at such a high temperature is mainly dominated by the ohmic resistance, which impedes the assessment of the difference in performance of the two perovskite electrodes. Nevertheless, both the ohmic and polarization resistance of the cell with L65SCrN perovskite were shown to be slightly decreased in comparison to the cell with stoichiometric electrode. This is also in accordance with a study observing the same trend by comparing stoichiometric L73SCrNi15 and A‐site deficient L63SCrNi15, although in the present study the difference is not as pronounced.^[8d]^ In the cited study, a significant increase in (electro‐)catalytic electrode activity of A‐site deficient perovskite electrodes was demonstrated at 800 °C. These effects were related to the increased oxygen vacancy formation caused by A‐site deficiency which enhanced the mobility of the lattice oxygen and increased Ni nanoparticle density on the surface. The authors reported a sixfold decrease in polarization resistance by employing an A‐site deficient perovskite. Although the compositions of the perovskites used in their and in our recent study are different,^[16]^ the behaviors in terms of thermogravimetric analysis (TGA) and XRD measurements were similar, showing 2.5 % oxygen weight loss at 900 °C upon reduction and the characteristic Ni peaks in the A‐site deficient perovskite materials. However, we could not observe a performance‐enhancing effect of similar magnitude. One reason could be that in the present work, the perovskite phase in L65SCrN is probably close to stoichiometry. An A‐site deficiency of less than 1 %, as discussed above is likely not to be sufficient to induce a significant enhancement over the L70SCrN. The difference in La/Sr ratio and in nickel concentration on B‐site among the two perovskites as estimated in the Supporting Information may also account for the small performance gain observed in the cell with L65SCrN electrode.

Another reason that not a larger difference between the two‐chromite based electrode is observed is the different operating temperatures between the present study (860 °C) and the study by Sun et al (800 °C).^[8d]^ The change of operating temperature could lead to a different role of Ni in the hydrogen oxidation mechanism. Two main effects of the formation of distributed metallic Ni particles on MIEC oxide anodes can be anticipated. First, it may facilitate dissociative adsorption of hydrogen.^[24]^ Second, it can favor the subsequent charge transfer reaction at the TPB that depending on the system, can either occur via hydrogen spillover, oxygen spillover or even via an interstitial bulk hydrogen transfer.^[25]^ For example, it has been indicated that for Ni/YSZ anodes adsorbed surface oxygen shows an intermediate stability on Ni and thus, facilitates the charge transfer kinetics according to the Sabatier principle.^[26]^ However, the latter effect only occurs if Ni‐bound intermediates actively take place in the rate‐limiting step (often the charge transfer reaction). If the charge transfer reaction occurs on the oxide surface at the perovskite/gas phase double phase boundary (DPB), Ni addition is not expected to have an influence on its kinetics. This was for example shown to be the case for Ni/CGO anodes when the CGO/gas phase DPB is large in comparison to the TPB.[^1a^, ^4c^] In such electrodes it was suggested that the rate‐limiting step occurs on the CGO surface without significant (electro‐)catalytic contribution of the Ni phase. Then the role of Ni is only to dissociate hydrogen and to provide electronic conductivity to the electrode. This means that in case of Ni cermet electrodes where hydrogen dissociation on the ceramic phase is sufficiently fast, Ni does not necessarily play an electro‐catalytic role during hydrogen oxidation.

The slightly increased performance of the L65SCrN electrode in comparison to L70SCrN could indeed indicate an increased surface area and, thus, increased availability of Ni active sites and their promoting effect on hydrogen oxidation kinetics. The presence of nickel particles originating from the NiO secondary phase likely did not cause the improvement of the polarization resistance because the resulting TPB length is likely far less than the one resulting from surface exsolved nanoparticles (see the Supporting Information). The only limited performance gain indicates that Ni does not actively contribute to the charge transfer step. The small performance gain could then suggest that hydrogen dissociation occurs on Ni but is probably not the rate‐limiting step under the investigated conditions. Possibly, this could rather be the charge transfer reaction occurring at the DPB between the perovskite and the gas phase after a hydrogen spillover from Ni. Since the operating temperature of 860 °C is high, it is possible that at lower temperatures they start to lead to more significant kinetic losses.

Since the kinetics are also dependent on the amount of Ni active sites, differences might become more apparent at lower temperatures. The change of rate‐limiting step during hydrogen oxidation on perovskites depending on temperature was also suggested by Zhu et al. who observed improved anode performance of Sr_0.95_(Ti_0.3_Fe_0.63_Ni_0.07_)O_3‐*δ*_ in comparison to the Ni‐free SrTi_0.3_Fe_0.7_O_3‐*δ*_ particularly at temperatures below 800 °C and low *p*H_2_.^[27]^ The authors proposed that Ni−Fe nanoparticle exsolution enhances hydrogen dissociative adsorption, which had been identified as key fuel oxidation rate‐limiting step at these conditions in a previous study.^[28]^ However, at conditions similar to the ones in the present study (850 °C, 97 % H_2_, 3 % H_2_O) the performance of both cells was similar.

In addition, it has been shown that in the case of La_0.8_Ce_0.1_Ni_0.4_Ti_0.6_O_3_ exsolved nanoparticles can also be submerged in the bulk of the host perovskite which can induce strain in the lattice and enhance oxygen transport.^[29]^ This bulk exsolution process seems to be particularly favored by A‐site deficiency and high operating temperatures, where the relatively high energy barriers associated with nucleation and growth within an oxide lattice that arise from strain and oxide lattice reconstruction around the particle can be overcome. Thus, providing that exsolution of nanoparticles may be favored in L65SCrN due to its composition and/or small A‐site deficiency it cannot be excluded that bulk and not only surface Ni particles contribute to the performance gain.

Nevertheless, the electro‐catalytic activity of both tested chromites reaches the one of a state‐of‐the‐art Ni/CGO anode at 860 °C although it is hard to clearly identify the origin of the small performance enhancement in the L65SCrN electrode.

To further demonstrate the influence of the different electrodes on cell performance, the ohmic overpotential of the Ni/CGO‐based cell was calculated by multiplying the ohmic resistance with the current density (Figure 2). The ohmic resistance was obtained from the Nyquist plot in Figure 4a and assumed to be constant over the whole investigated potential range. By subtracting the obtained values from the OCV, the *i*‐*V* curve of an ideal non‐polarizable electrode can be calculated. This has been done for the Ni/CGO‐based cell since metallic nickel is an excellent electronic conductor and thus, Ni/cermet electrodes are usually assumed to not display any losses caused by electronic resistivity. Thus, in Figure 2 the *i*‐*V* curve of a cell with two ideal non‐polarizable electrodes, that is, no anode/cathode resistance and also no mass transport resistance, is depicted. The difference between this hypothetical curve and the actually measured *i*‐*V* curves can then be ascribed to the polarization resistance of the cell. The graph clearly demonstrates that all cells’ performance behavior is strongly dominated by the ohmic overpotential. Considering the favorable electrochemical performance of the 25 μm thick L65SCrN and 19 μm thick L70SCrN perovskite electrodes (based on electrochemical impedance spectra), the difference between their *i*‐*V* curves and the one of Ni/CGO can almost exclusively be attributed to their larger ohmic losses and thus, to their limited electronic conductivity.

The comparatively small difference of 0.18 A cm^−2^ at 0.6 V between the polarization curve of the Ni/CGO‐based ESC (0.98 A cm^−2^) and the hypothetical curve with two ideal non‐polarizable electrodes (1.16 A cm^−2^) illustrate the potential improvement in cell performance by further optimizing the electrode kinetics of the anode. If electrocatalytic activity of the LSCrN based electrode is already sufficient under the given conditions (860 °C), improvement of current collection in the LSCrN based electrode represent the key challenge to match performance of state‐of‐the‐art Ni/CGO anodes.

### Sulfur poisoning effect

**Figure 6:**
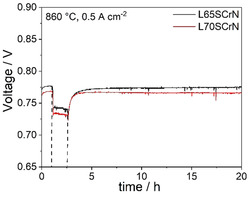
Transient sulfur poisoning test at 860 °C and 0.5 A cm^−2^ for cells with 25 μm thick L65SCrN and 19 μm thick L70SCrN electrode. After 1 h in sulfur‐free conditions, 20 ppm H_2_S were introduced. After 1.5 h, the sulfur supply was switched off again.

As a next step, the performance of two cells with 25 μm thick L65SCrN and 19 μm thick L70SCrN electrode was assessed at 0.5 A cm^−2^ with and without the addition of 20 ppm H_2_S in the fuel gases. The evolution of the voltage over time during the poisoning tests is depicted in Figure 6.

**Figure 7:**
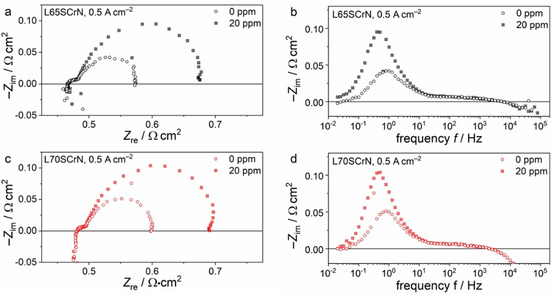
Complex‐plane and imaginary impedance plot of the electrochemical impedance spectra of the cells with 25 μm thick (a,b) L65SCrN and (c,d) 19 μm thick L70SCrN electrode with and without the addition of 20 ppm H_2_S.

Consistent with the *i*‐*V* curves in Figure 2, the initial voltage of 0.777 V of the cell with L65SCrN electrode is slightly higher than the 0.769 V observed for the L70SCrN electrode. After 1 h of voltage stabilization, 20 ppm H_2_S were introduced into the fuel gas. This led to the nearly same rapid voltage drop of 35 and 34 mV for the L65SCrN and the L70SCrN electrode, respectively. After 1.5 h of poisoning, the cells were recovered by switching off the sulfur supply. This led to a nearly full regeneration of the initial voltage after 15 h. The effect of the sulfur poisoning on the impedance spectra at 0.5 A cm^−2^ is shown for the cells with L65SCrN and the L70SCrN electrode in Figure 7. Both cells show the same behavior of a pronounced increase in polarization resistance related to the anode contribution at approximately 1 Hz with no change of the ohmic resistance.

The observed pronounced sensitivity of the electrode to sulfur poisoning is in agreement with our recent observations about Ni/CGO and Ni/YSZ anodes, where we could correlate the rapid voltage drop with Ni surface blockage by adsorbed sulfur atoms.[^1^, ^30^] Therefore, it is reasonable to assume a comparable phenomenon for the exsolved Ni nanoparticles on the perovskite surface. The voltage drops of approximately 35 mV at 860 °C in the present study are significantly lower than the values of more than 150 mV observed for Ni/YSZ at 850 °C at the same current density and gas phase conditions, which demonstrates the superior tolerance of LSCrN electrodes compared to Ni/YSZ cermets. However, this is still 3.5 times higher than the values we observed for Ni/CGO cermets.^[1a]^ Therefore, considering the above‐mentioned anode materials in ESC configuration, the ranking regarding their tolerance towards sulfur poisoning is Ni/CGO>LSCrN≫Ni/YSZ.

Although we suggested the hydrogen dissociation not to be rate‐limiting at 860 °C in sulfur‐free hydrogen, this might change upon addition of 20 ppm H_2_S which will lead to an estimated sulfur coverage of 0.83 on Ni according to the Temkin‐like isotherm derived by Alstrup et al.^[31]^ This will block the Ni surface to a large extent for hydrogen adsorption and dissociation and, thus, lead to an increase of the anode polarization resistance.

Interestingly, Sun et al. observed a promoting effect of 5000 ppm H_2_S on hydrogen oxidation at 800 °C.^[8d]^ They suggested that the hydrogen sulfide may adsorb on the surface oxygen site of the perovskite to form a H_2_−S−O bond that is an effective carrier for H_2_ to react with oxygen ions since the H−S bond might be easier to break than the H−H bond. Such a promoting effect could not be observed in our study. At the significantly lower H_2_S content that we have used, it is likely that the observed performance drop is mostly due to the deleterious effect of the formation of the Ni−S bond on the surface of the exsolved nickel particles. Thus, the different behavior would be due to a different nature of the sulfur‐electrode interaction that might become favorable at higher sulfur concentrations and/or lower temperature. Indeed, at typical SOFC operating temperatures and low H_2_S concentrations sulfur does not only adsorb on the Ni surface but reacts to Ni sulfide compounds at concentrations of 1000 ppm or higher.^[8d]^

### Durability and redox stability of L65SCrN

Durability and redox stability were investigated with the L65SCrN anode that reached a higher cell performance than L70SCrN (see Figure 2). Cells with 8 μm and 25 μm thick LSCr65N anodes were compared in order to further understand their behavior (Figure 2).

#### Cell with 8 μm thick anode

**Figure 8:**
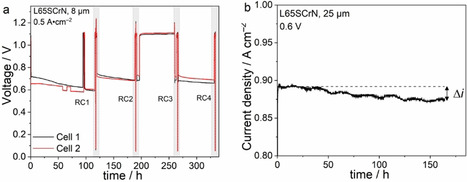
(a) Life cycle of the cell with 8 μm thick L65SCrN anode with indication of the RC. (b) Durability test of the cell with 25 μm thick L65SCrNat 0.6 V. All measurements were performed at 860 °C.

Figure 8a shows the voltage evolution of two nominally equal cells with 8 μm thick anode that were operated at 0.5 A cm^−2^ exposed to several redox cycles (RC) by purging the anode chamber with air for 45 min. The testing protocol of the cells is reported in Table 1. In the protocol, cell 2 went through four redox cycles, whereas cell 1 only through one. For both cells, the time under polarization led to a severe voltage degradation that has been recovered after each redox cycle.

**Table 1:** Protocol of redox cycling test of the two cells with 8 μm thick L65SCN electrodes.

Time period	Testing conditions	
[h]	Cell 1	Cell 2
<0	18 h reduction in H_2_	
0–94	Constant current density of 0.5 A cm^−2^	
94–97	OCV (current cycle)	
97–116	Constant current density of 0.5 A cm^−2^	
117	**Redox cycle 1**	
120–188	Constant current density of 0.5 A cm^−2^	
189	OCV, 5 % H_2_, 95 % N_2_	**Redox cycle 2**
192–196	Constant current density of 0.5 A cm^−2^	
197–259	OCV, 5 % H_2_, 95 % N_2_	
260–264	Constant current density of 0.5 A cm^−2^	
265	OCV, 5 % H_2_, 95 % N_2_	**Redox cycle 3**
266–231	Constant current density of 0.5 A cm^−2^	
332	OCV, 5 % H_2_, 95 % N_2_	**Redox cycle 4**
333	Cool‐down, 5 % H_2_, 95 % N_2_	

**Figure 9:**
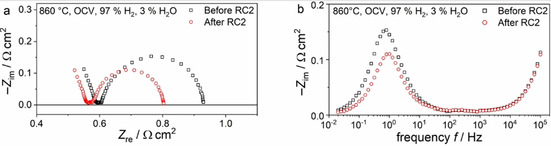
(a) Complex‐plane and (b) imaginary impedance plots of the electrochemical impedance spectra of the cells with 8 μm thick L65SCrN before and after RC 2.

**Figure 10:**
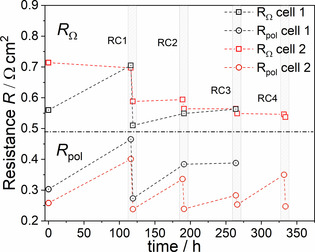
Evolution of ohmic and polarization resistance of the cells with 8 μm thick L65SCrN electrodes during 4 RC.

The effect of a redox cycle on the cell impedance spectra is exemplified in Figure 9 for cell 2. After the second redox cycle impedance spectra show both a reduced ohmic and polarization resistance. In the polarization resistance the peak frequency of the affected process lies at approximately 1 Hz confirming the assignment of this frequency region to an anode process as suggested in the previous subsection. A breakdown of the cell resistance before and after each redox cycle into ohmic and polarization losses for both cells is depicted in Figure 10.

Between the initial reduction and the first redox cycle after 117 h, both cells showed significant degradation. In this period, the cell voltages decreased by 128 and 50 mV for cell 1 and cell 2, respectively. Nevertheless, the observed voltage drops may have different origins. For cell 1 this was nearly equally due to an increase of the ohmic and the polarization resistances. Both contributions were entirely recovered to their initial values after redox cycle 1. For cell 2, on the contrary, the voltage decrease was almost entirely attributed to a significant increase in polarization resistance, while the ohmic resistance even decreased slightly in the first 117 h. Despite different initial values of *R*_ohm_ for cell 1 and cell 2, they converged to a comparable value of approximately 0.7 Ω cm^2^ after 117 h, and the performance of both cells was comparable with a voltage of 0.741 and 0.716 V at 0.5 A cm^−2^ for cell 1 and cell 2, respectively. Beyond this time, the ohmic resistance evolved differently for the two cells. Cell 1 achieved the lowest *R*_ohm_ value after RC1, while the value continuously deteriorated with time afterwards. For cell 2 on the contrary, the *R*_ohm_ value improved with each occurrence of a redox cycle, so that the lowest value was measured after RC4. This difference in behavior could be assigned to a specific evolution of the contacting at the oxygen electrode and its interface with the gold mesh due to creeping at high temperature as we already observed in previous experiments in our testing set up. This effect led to a better contacting of the cells over time and thus, a decrease in ohmic resistance. The effect was more pronounced for cell 2 explaining its lower initial performance and smaller initial degradation rate. Thus, it is likely that a faulty contacting of cell 2 accounted for the difference in evolution of the *R*_ohm_ between the two samples.

Redox cycles 2, 3, and 4 were only carried out for cell 2. In between each cycle the cell operation mainly led to an increase in polarization resistance and an only minor increase in ohmic resistance. After each new redox cycle, the degradation was fully recovered. The degradation rate of the polarization resistance in each step was similar when the cell was operated at 0.5 A cm^−2^ (Δ*R*_pol0,1_=0.123 Ω cm^2^/100 h; Δ*R*_pol1,2_=0.14 Ω cm^2^/100 h; Δ*R*_pol3,4_=0.147 Ω cm^2^/100 h). However, it significantly reduced when the cell was left at OCV between redox cycle 2 and 3 (Δ*R*_pol2,3_=0.06 Ω cm^2^/100 h). Since the fuel utilization at 0.5 A cm^−2^ was only 6 % and thus, the change in steam content in the fuel gas compartment was low, these findings suggest the anodic bias to be the reason for the increased degradation rate under current.

**Figure 11:**
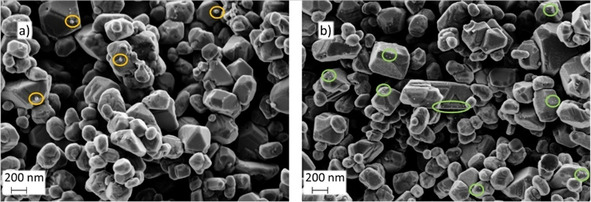
SEM images of the fractured 8 μm thick L65SCrN electrodes of the (a) degraded cell (b) and the cell that was exposed to a redox cycle before cool‐down. (a) Orange and (b) green circles are shown to highlight the nanoparticles.

In order to correlate the influence of redox cycling on cell performance with changes in the L65SCrN electrode material, cell 2 was recovered by exposure to RC4 before cooling down, while cell 1 had been operated for more than 200 h after the last redox cycle and was cooled down without redox cycling. Both cells were cooled down in forming gas with 5 % H_2_ and 95 % N_2_. Figure 11 shows a comparison of the two fracture surfaces.

The degraded cell 1 (Figure 11a) shows a few nanoparticles of approximately 50 nm diameter on top of the perovskite surface (orange circles). However, the recovered cell 2 (Figure 11b) depicts a significantly greater amount of Ni nanoparticles of approximately 10–20 nm particle size.

The presence of fewer nanoparticles may indicate a change in the oxidation state of the perovskite due to the anodic bias in SOFC operation, which probably leads to changes in the defect chemistry of the perovskite. This could have a significant effect on the perovskite's transport properties and surface chemistry, including the presence of Ni nanoparticles. Ni exsolution occurs via the reduction of Ni from the (+II or +III) state to a neutral charge [Eq. 2]:

(2) $$\mathrm{Ni}{}^{n+}+ne{}^{-}\;\vec{\leftarrow }\;\mathrm{Ni}{}^{0};\;n=2,\;3$$

where the electrons e^−^ are supplied by the oxygen contained in the perovskite upon the reaction with hydrogen. Thus, the electron loss may counteract the chemical reduction of the anode at OCV (860 °C with 3 % H_2_O/H_2_) and favor the formation of Ni^2+^/Ni^3+^ with a subsequent re‐incorporation of Ni nanoparticles into the perovskite lattice as Ni cations.

Similarly fast degradation as for the 8 μm thick L65SCrN electrode has been reported in literature for stoichiometric LSCrN perovskite anodes and was correlated with Ni agglomeration.[^8a^, ^13^] Exsolved Ni particles agglomerated on a chromite anode at 800 °C and OCV over 300 h, while Ru particles were more stable. This behavior was suggested to be caused by the high surface diffusivity of Ni in comparison to Ru metal particles on chromite surfaces. The nanoparticle size was also reported to increase with reduction time for other parent perovskites.^[32]^ The presence of larger nanoparticles on the degraded electrode in Figure 11a is consistent with this proposed particle growth mechanism. It is even possible that particle agglomeration and incorporation under anodic bias occur simultaneously.

The presence of numerous small nanoparticles in Figure 11b indicates their regeneration by redox cycling, which correlates well with the performance recovery observed after each redox cycle. This is also in agreement with previous studies, that suggested Ni reincorporation into the chromite lattice upon oxidation and exsolution back to the surface as small, well‐dispersed metallic particles after every reduction step.[^8d^, ^8e^, ^10a^] Such fully reversible exsolution and dissolution after redox cycling has recently been observed by transmission electron microscopy (TEM), energy‐dispersive X‐ray spectroscopy (EDX), and XRD for CoFe alloy nanoparticles in La_0.4_Sr_0.6_Co_0.2_Fe_0.7_Mo_0.1_O_3‐*δ*_ and Co‐doped Sr_2_Fe_1.5_Mo_0.5_O_6‐*δ*_.[^32^, ^33^] In a few publications, it was suggested that exsolved particles do not necessarily dissolve back into the bulk, and are still present on the surface in their oxidized state.^[34]^ However, this was mainly observed between 500–700 °C and probably related to the sluggish kinetics at such low temperatures. Furthermore, the oxidized particles could still be observed by scanning electron microscopy (SEM). Based on the SEM analysis in Figure 11 we report that it is likely that Ni nanoparticles in the present work can be re‐incorporated into the lattice.

However, compared to what we observed recently on L65SCrN powders reduced at 900 °C,^[16]^ the sample exposed to a redox cycle before cooling showed a significantly reduced population density of Ni nanoparticles. The phase transformation of Ni exsolution has been shown to proceed with prolonging time,[^8b^, ^32^] and since the redox‐cycled electrode was only operated in hydrogen for 1 h before cooling down, it is possible that equilibrium had not been reached.

In any case, the observed increased degradation under anodic bias contrasts to the operation of electrodes with extensive Ni exsolution in electrolysis mode, where Ni exsolution was shown to be favored since the equilibrium of Equation (2) is shifted to the right side.

**Figure 12:**
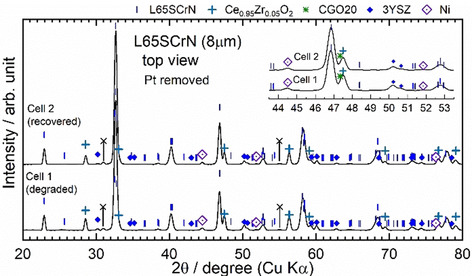
XRD patterns of the 8 μm thick L65SCrN electrodes of the degraded cell, and the cell that was exposed to a redox cycle before cool‐down.

In addition to SEM/EDX analysis, the two electrodes were characterized by XRD. For this purpose, the Pt current collectors were scratched off and the samples were analyzed from a top view perspective (Figure 12). All the layers down to the 3YSZ electrolyte were identified by XRD with only minor unknown peaks (marked with X around 31 and 55°). A possible candidate of this minor phase is the La_2_Sr_2_PtO_7_ perovskite, however, it is not yet clearly identified.^[35]^ No significant differences could be observed in the diffraction patterns of the two samples. Therefore, it can be excluded that large‐scale phase transformations of the L65SCrN electrode occur during SOFC degradation that are reversed by redox cycling. The observed perovskite phase corresponds to the one observed in the powder in Figure 1 and metallic Ni was detected in both samples, which is an overlap of the Ni that stems from the initial NiO secondary phase and the nanoparticles formed on the surface and in the bulk. Since the intensity and width of these peaks in both samples are similar, no quantitative difference can be reported. The characteristic peaks of two fluorite phases were observed, which correspond to the 3YSZ and CGO20 layers. The peaks of the CGO20 layers are shifted to slightly higher angles than the cubic fluorite CGO20 phase (PDF#01‐080‐5535). This lattice shrinkage can be explained by the formation of an interdiffusion zone between CGO and YSZ that results in a solid solution phase of ceria and zirconia.^[36]^

In any case, the large differences in morphology from our recent study suggests that based on a post mortem analysis of the perovskite surfaces by SEM, their in situ state cannot be inferred.^[16]^ Thus, no detailed information about possible microstructure‐related degradation phenomena such as Ni coarsening or Ni re‐integration into the lattice can be given. More detailed experiments will be carried out in the future to further explore the observed effect.

#### Cell with 25 μm thick anode

Figure 8b shows a potentiostatic durability test at 0.6 V over 175 h for a cell with the 25 μm thick electrode. The cell only shows a current density decrease of 19 mA cm^−2^ from 0.893 to 0.874 V over the entire course of the experiment. Although the operating conditions were different from the test of the cells with 8 μm thick anodes, this 2 % voltage decrease over 175 h represents a significantly lower performance loss than the ones observed for the cells with thinner electrodes. After the 175 h, the cell was exposed to a redox cycle and then cooled down. The cell did not show a recovery effect upon redox cycling which suggests a different origin of its degradation that seems to be irreversible in contrast to the one observed for the cells with 8 μm thick anodes.

Based on the observed influence of anode overpotential on performance degradation for the 8 μm thick electrode and the impact of the current restriction on L65SCrN electrode performance, it is likely that the combination of these effects is responsible for the increased stability of the 25 μm thick electrode.

We showed L65SCrN degradation in the 8 μm thick electrode to be strongly potential‐dependent with more severe degradation at lower cell potential. However, as discussed above the 8 μm thick electrode is operated at significantly larger anode overpotential due to the current restriction effect. Therefore, the 8 μm thick electrode shows a reduced effective electrochemically active surface area and a larger current density per effective electrochemically active surface area. This leads to a reduced local potential step (i. e., increased local overpotential) in the electrochemically active area which entails increased degradation. According to this hypothesis, it is likely that the decreased stability of the 8 μm thick L65SCrN electrode can be explained by a less homogeneous current distribution along the cell. According to Equation (2) metallic Ni is less stable if the electrode is operated at a low cell potential, that is, high anode overpotential, and it is possible that at such conditions the exsolved Ni is re‐incorporated into the perovskite lattice as Ni^2+^/Ni^3+^. Alternatively, the strongly potential‐dependent degradation behavior could possibly be due to enhanced Ni particle coarsening at high anode overpotentials.

## Conclusions

Ni‐doped chromite anodes were successfully integrated into electrolyte‐supported cells (ESC) with 5×5 cm^2^ size and their performance, durability, redox stability, and sulfur tolerance was investigated in solid oxide fuel cell (SOFC) operation with H_2_/H_2_O fuel gas. In comparison to infiltration techniques that lead to rapid performance losses due to fast particle agglomeration, the present study uses an industrially more viable approach to incorporate Ni nanoparticles into the anode via exsolution. Ni nanoparticles are formed on the perovskite surface in situ upon reduction. In comparison to the stoichiometric La_0.7_Sr_0.3_Cr_0.85_Ni_0.15_O_3‐*δ*_ (L70SCrN) electrode, a nominal La_0.65_Sr_0.3_Cr_0.85_Ni_0.15_O_3‐*δ*_ (L65SCrN)‐based cell showed slightly better performance due to previously observed, enhanced Ni exsolution. Cells with both chromite anodes showed promising performance approaching the ones of state‐of‐the‐art Ni/gadolinium‐doped ceria (Ni/CGO) anode‐based cells. The difference was shown to originate from an increased ohmic resistance of the chromite electrodes, while similar polarization resistance values were observed. 25 μm thick L65SCrN electrodes exhibited both superior performance and stability in comparison to 8 μm thick electrodes. The difference can be explained by the current restriction effect in the thinner anode. The chromite‐based anodes showed a strongly potential‐dependent degradation behavior that is likely related to Ni nanoparticle coarsening on the perovskite surface, or Ni re‐integration into the lattice. Redox cycling led to recovery of the anode degradation due to re‐integration of the Ni particles into the perovskite lattice. Sulfur poisoning with 20 ppm hydrogen sulfide led to rapid voltage drops of 35 mV at 0.5 A cm^−2^. Discussion of the mechanism leads to the conclusion that Ni nanoparticles facilitate hydrogen dissociation to the extent that it is not rate‐limiting at the investigated temperature unless an insufficiently thick electrode thickness is employed or sulfur impurities are present in the feed gas. Hence, approaches for future research could be the increase of the electronic conductivity of Ni‐based chromite anodes by tuning the phase composition or by adding a highly electronically conductive second phase. At the high operating temperatures of 860 °C used in this study, the electrocatalytic activity of LSCrN anodes with Ni exsolution is sufficient to compete with state‐of‐the‐art Ni/CGO electrodes. However, if lower operating temperatures are targeted, the performance of the perovskite anodes should be re‐evaluated.

## Experimental Section

### LSCrN powder crystallographic characterization

La_0.65_Sr_0.3_Cr_0.85_Ni_0.15_O_3‐*δ*_ (L65SCrN) and La_0.7_Sr_0.3_Cr_0.85_Ni_0.15_O_3‐*δ*_ (L70SCrN) powders were supplied by Marion Technologies (Verniolle, France) with particle size diameters of approximately 300–500 nm. To ensure compatibility with our previous study,^[16]^ the crystalline structure of both commercial powders were analyzed (before and after reduction) by XRD using a D8 ADVANCE (BRUKER AXS GmbH, Germany) diffractometer with a CuK_α_ radiation source operating at 40 kV and 40 mA in Bragg‐Brentano geometry. A variable divergence slit (12 mm) was used for the primary optics and soller slits (2.5°) were used for both the primary and secondary sides. Diffraction signal was collected by the LYNXEYE XE‐T in 2*θ* range of 10–135° with a scanning rate of 0.4 s per step and an increment step of 0.02°. High energy resolution mode was used on the LYNXEYE XE‐T to filter the fluorescent emission from Ni.

### Cell manufacturing

L65SCrN and L70SCrN inks were prepared by dispersing the perovskite powder in a 94 wt % α‐terpineol and 6 wt % ethyl cellulose solution with a powder/solution ratio of 2 : 1 and then mixing in a 3‐roll mill. Subsequently, different ESC were manufactured by using a commercial square substrate (5×5 cm^2^ and 90 μm thickness) of 3 mol% Y_2_O_3_‐doped ZrO_2_ electrolytes coated on both sides with Ce_0.8_Gd_0.2_O_2‐*δ*_ (CGO20) from Kerafol GmbH (Eschenbach, Germany). As anodes, 4×4 cm^2^ L65SCrN and L70SCrN perovskites were implemented via screen‐printing. Two different mesh thicknesses were used for the L65SCrN anode to obtain different electrode thicknesses of approximately 8 and 25 μm (after firing). For the L70SCrN anode, only the latter mesh was using during the screen‐printing to produce an electrode with a thickness of 19 μm. The resulting half cells were fired at 1200 °C for 1 h in air with a heating rate of 3 K min^−1^. Afterwards, the cathode was screen‐printed on the other side of the electrolyte with a commercial ink of La_0.58_Sr_0.4_Fe_0.8_Co_0.2_O_3‐δ_ (LSCF) supplied by Heraeus GmbH (Hanau, Germany) resulting in a cathode thickness of approximately 20 μm. Pt paste was brushed on the sintered anode surface for current collection. Then, the cell was fired at 1050 °C for 1 h in air with a heating rate of 3 K min^−1^.

Furthermore, state‐of‐the‐art cells supplied by Sunfire GmbH (Dresden, Germany) were used as a reference. They are based on the same CGO20(5 μm)|3YSZ (90 μm)|CGO20 (5 μm) sandwich structure as the cells in the present work. A Ni/CGO20 anode was deposited including a functional layer and a more porous current collector layer with increased Ni content. On the cathode side, a LSCF/CGO composite cathode with a LSCF current collector layer was used.

### Electrochemical characterization

The setup for cell testing enables the characterization of up to four cells simultaneously under variation of current density and has been illustrated and described in detail elsewhere.[^1a^, ^30^] The high reproducibility of measurements between the different positions has been demonstrated in previous studies.[^1b^, ^18^, ^37^] All cells were operated at 860 °C with 97 % H_2_ and 3 % H_2_O at a constant total fuel flow rate of 1 L min^‐1^ for every cell. H_2_S was taken from a pressurized H_2_S/H_2_ bottle that contained 200 ppm H_2_S. The cathode was operated with air at a constant flow rate of 1 SLPM. The cells were heated (3 K min^−1^) to 900 °C for sealing and reduction. Proper sealing of all cells was ensured by confirming the OCV to be higher than 1.2 V in pure hydrogen. Pt and Au meshes were used for contacting on the anode and the cathode side, respectively.

Electrochemical impedance spectroscopy (EIS) was performed by means of an electrochemical workstation (Zahner® PP‐240 with Thales software) in a frequency range from 20 mHz to 100 kHz with 10 points per decade. The amplitude of the current stimulus was chosen to be 500 mA. SEM images were acquired using a Zeiss Ultra Plus SEM. XRD analysis of the cells was also performed after the test operation using D8 Discover GADDS equipped with a VÅNTEC‐2000 area detector (Bruker AXS, Germany). The diffraction pattern was recorded in the Bragg‐Brentano geometry with a tuned monochromatic and collimated CuK_α_ radiation source.

## Conflict of interest

The authors declare no conflict of interest.

## Supporting information

As a service to our authors and readers, this journal provides supporting information supplied by the authors. Such materials are peer reviewed and may be re‐organized for online delivery, but are not copy‐edited or typeset. Technical support issues arising from supporting information (other than missing files) should be addressed to the authors.

Supplementary
