# Supplementary material for: Performance and Limitations of Nickel‐Doped Chromite Anodes in Electrolyte‐Supported Solid Oxide Fuel Cells
Source: ChemSusChem. 2021 May 7;14(11):2401–13. doi: 10.1002/cssc.202100330 (PMC8252760; doi:10.1002/cssc.202100330)
Supplement: Supplementary file 1 — Supplementary [file CSSC-14-2401-s001.pdf]

ChemSusChem

Supporting Information

Performance and Limitations of Nickel-Doped Chromite Anodes in Electrolyte-Supported Solid Oxide Fuel Cells

Matthias Riegraf,* Diana M. Amaya-Dueñas, Noriko Sata, K. Andreas Friedrich, and Rémi Costa*© 2021 The Authors. ChemSusChem published by Wiley-VCH GmbH. This is an open access article under the terms of the Creative Commons Attribution License, which permits use, distribution and reproduction in any medium, provided the original work is properly cited.

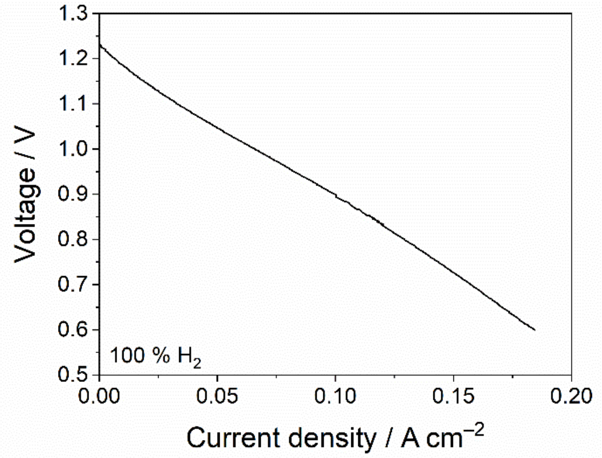

Fig. S1. Current-voltage characteristics of a cell with a brushed Pt layer as functional fuel electrode.

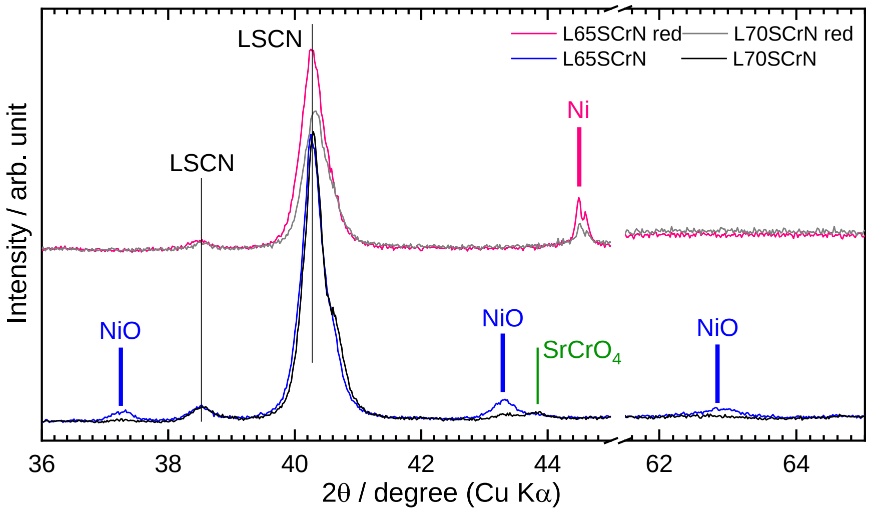

Fig. S2. XRD patterns of the reduced and as-prepared L65SCrN and L7SCrN powder.

Rietveld quantification

A Rietveld refinement was carried out with the TOPAS software using XRD measurements from the Bruker D8 Advance.

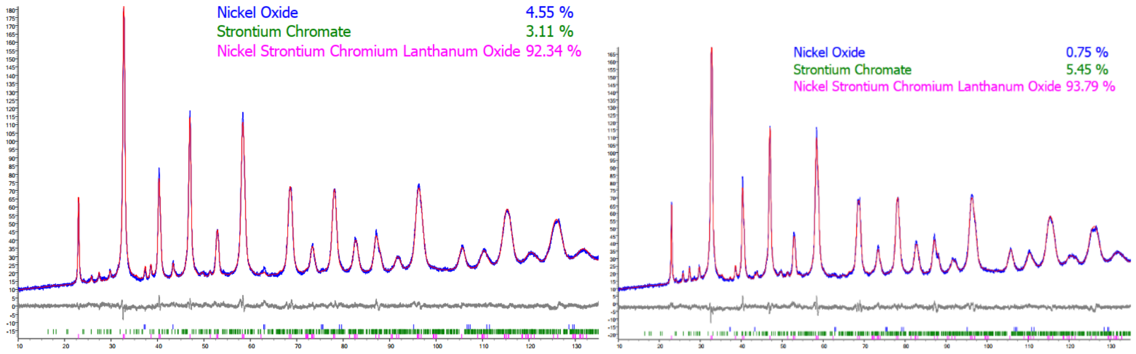

Fig. S3: Rietveld refinement of the raw L65SCrN (left) and L7SCrN (right) powder.

Based on the Rietveld refinement and the quantified phases in the supplementary information, an estimation of the composition (A/B ratio, Sr content on A site, Ni content on B site) of the L65SCrN and L7SCrN perovskites can be made:

<u>L65SCrN:</u>

$La_{0.65}Sr_{0.3-0.031}Cr_{0.85-0.0311}Ni_{0.85-0.0455}O_{3-\delta}$ =

$La_{0.65}Sr_{0.2689}Cr_{0.8189}Ni_{0.1045}O_{3-\delta}$

A/B ratio: 0.995

Sr content on A site: 0.29

Ni content on B site: 0.11

<u>L7SCrN:</u>

$La_{0.7}Sr_{0.3-0.0545}Cr_{0.85-0.0545}Ni_{0.85-0.0075}O_{3-\delta} = La_{0.7}Sr_{0.2455}Cr_{0.7955}Ni_{0.1425}O_{3-\delta}$

A/B ratio: 1.008

Sr content on A site: 0.26

Ni content on B site: 0.15

The ratio between moles on the A-site and the B-site in O L65SCrN is 0.995, corresponding to an estimated A-site deficiency of 0.5 mol%.

Estimation of TPB lengths

The approximate TPB length of the Ni/L65SCrN/gas phase interface can be calculated based on the assumption that all Ni particles are perfect spheres with the mean diameter *d:*

$TPB = \pi * d * N = \pi * d * x_{Ni} * M / (V*\delta) = 6 * x_{Ni} * M / (d^2 * \delta)$,

with *m* being the mass, *V* the volume, *M* the molar mass and δ the density of Ni, and x_{Ni} the molar fraction of Ni.

To determine the TPB length contributions of Ni particles originating from the reduced NiO secondary phase and exsolved Ni particles, the respective values can be estimated. The reduced NiO particles are expected with a size in the sub-µm / µm range. A rather small particle size diameter of 0.25 µm was assumed. The exsolved Ni nanoparticles are much smaller as shown in Fig. 11 and were assumed to have an average diameter of 20 nm. For a molar fraction $x_{Ni,submicron}$ of 0.0455, corresponding to the amount of NiO in the oxidized powder, a TPB length of 2.67 10^{13} µm/mol is obtained. When only 0.6 mol % of Ni is exsolved, a value taken from our recent study of the same material (Amaya-Dueñas et al., *J. Mat. Chem. A* 2021, 9, 5685), a TPB length of $5.93*10^{14}$ µm/mol is obtained. The more than one order of magnitude larger TPB length corresponding to the exsolved Ni indicates that the influence of exsolved Ni is dominating the electro-catalytic contribution of the Ni phase in the L65SCrN electrode. This contribution is probably even larger for the L7SCrN electrode due to the lower amount of NiO in the raw powder.
